# Prevalence and Cognitive Profiles of Children With Comorbid Literacy and Motor Disorders

**DOI:** 10.3389/fpsyg.2020.573580

**Published:** 2020-12-11

**Authors:** Cameron Downing, Markéta Caravolas

**Affiliations:** ^1^School of Psychology, Bangor University, Bangor, United Kingdom; ^2^Miles Dyslexia Centre, Bangor University, Bangor, United Kingdom; ^3^Department of Social and Behavioural Sciences, Leeds Trinity University, Leeds, United Kingdom

**Keywords:** comorbidity, dyslexia, developmental coordination disorder (DCD), prevalence, cognitive profiles, multiple deficit model

## Abstract

There is a high prevalence of comorbidity between neurodevelopmental disorders. Contemporary research of these comorbidities has led to the development of multifactorial theories of causation, including the multiple deficit model (MDM). While several combinations of disorders have been investigated, the nature of association between literacy and motor disorders remains poorly understood. Comorbid literacy and motor disorders were the focus of the two present studies. In Study 1, we examined the prevalence of comorbid literacy and motor difficulties relative to isolated literacy and motor difficulties in a community sample (*N* = 605). The prevalence of comorbidity was five times greater than expected by chance alone, implying some relationship between difficulties. In Study 2, we examined the cognitive profiles of children with literacy and motor disorders amongst a subsample of children from Study 1 (*N* = 153). Children with literacy disorder had deficits in phonological processing, selective attention, and memory whilst children with motor disorder had deficits in visuospatial processing and memory, suggesting the disorders should be considered to have both independent and shared (memory) cognitive risk factors. Children with comorbid literacy and motor disorder demonstrated an additive combination of these deficits. Together, these findings are consistent with predictions from the MDM.

## Introduction

Disorders of literacy such as dyslexia, and of motor skills such as developmental coordination disorder (DCD), are complex, behaviorally defined, and neurodevelopmental in origin. Dyslexia is a disorder affecting accurate and fluent word reading and spelling (Rose, 2009), and DCD is a disorder affecting the acquisition and execution of coordinated motor skills (Blank et al., 2019). Despite being disorders of separate domains, it is reported that they are frequently comorbid with one another (Kaplan et al., 1998), and a notable overlap of cognitive impairments between these conditions is often reported. However, supporting evidence is scant. In this paper, we test whether the multifactorial view of neurodevelopmental disorders (e.g., Pennington, 2006) adequately explains comorbidity between literacy and motor disorders by (a) establishing whether the prevalence of comorbid literacy and motor difficulties is greater than expected and (b) investigating the nature of cognitive deficits in literacy, motor, and comorbid literacy and motor disorders.

The current view is that the etiology of neurodevelopmental disorders is multifactorial in nature (Thapar and Rutter, 2015). Accordingly, Pennington (2006) proposed the multiple deficit model (MDM) which conceptualizes disorders over four levels. At the etiological level, complex interactions between environmental and genetic risk and protective factors influence the development of multiple neural systems, either at the same time, or successively during later development. Neural systems affect the development and action of multiple cognitive processes which interact with one another. The impairments at the cognitive level lead to behavioral impairments at the disorder(s) symptom level.

An advantage of this model over alternative single-deficit models is that it offers a holistic and parsimonious explanation of the highly comorbid nature of neurodevelopmental disorders. Several different hypotheses about comorbidity assume that each disorder arises from a single underlying cause. These single-deficit explanations include the *severity hypothesis* (the deficits are associated with disorder *a* and a comorbid disorder *b*, but are separable to the deficits of disorder *b*), *synergy hypothesis* (separate deficits are associated with disorders *a* and *b*, but comorbidity between *a* and *c* leads to disorder *b*, although disorder *b* can also develop from other deficits), *cross-assortment hypothesis* (separate deficits for disorders *a* and *b*, but those with either disorder are more likely to have offspring with an individual with the other disorder), *pleiotropy hypothesis* (a single etiology manifests in two separate cognitive deficits which lead to separate disorders but can co-occur in comorbid cases), and *genetic heterogeneity hypothesis* (separate etiologies manifest in one cognitive deficit leading to comorbid cases). Pennington (2006) argues that none of these hypotheses adequately explains the independent and shared aetiological nature of the comorbidity between dyslexia and speech sound disorder. Rather, only a multiple deficit explanation may adequately explain comorbidity. Indeed, evidence for this multifactorial account of comorbid disorders has also been found in investigations of heterotypic comorbidities, namely in explaining the comorbidity between dyslexia and ADHD (McGrath et al., 2019). Investigations have reported shared genetic risk (e.g., Willcutt et al., 2002) and cognitive deficits (e.g., Gooch et al., 2011) amongst children with dyslexia, ADHD, and comorbid dyslexia and ADHD in both clinic- and community-based samples (Germanò et al., 2010).

We present two related studies, which seek to test whether the MDM adequately explains comorbidity between a disorder of word-level literacy (consistent with dyslexia) and motor skills (consistent with DCD). In the first study, we tested a key prediction of the multiple deficit model (MDM; Pennington, 2006) that the incidence of comorbid literacy and motor disorders is greater than expected based on the rates of isolated disorders. In the second study, we examined the cognitive profiles of children with literacy, motor, and comorbid literacy and motor difficulties, using subsamples from Study 1. In this latter study, we sought to identify shared and independent risk factors of literacy and motor disorders. In addition, we investigated the profiles of children with comorbid literacy and motor disorders to better understand the nature of their comorbidity. We tested three competing behavioral-genetic hypotheses which have been used to test the nature of comorbidity between dyslexia and ADHD (de Jong et al., 2006), but could be readily applied to test the comorbidity between literacy and motor disorders. These hypotheses are phenocopy (the etiology associated with one disorder manifesting as a second disorder), cognitive subtype (the etiology of comorbid disorders is distinct to that of the isolated disorders), or shared etiology hypothesis (there is some common etiology between the disorders). The phenocopy and cognitive subtype hypotheses attempt to account for comorbidity following a single deficit explanation whereas the shared etiology hypothesis follows a multiple deficit account.

## Study 1

A crucial step in examining the relationship between disorders is to determine whether the frequency of comorbid disorders is greater than that predicted from the base rates of isolated disorders. If the frequency of children with comorbid disorders is greater than the frequency predicted from the combined frequency of isolated disorders, it can be concluded that comorbidity is not the result of statistical chance. Rather, it is likely that the two disorders are related. However, analyzing the frequency of comorbid disorders in clinic-based samples leads to artificially inflated prevalence estimates (see Caron and Rutter, 1991). To assess whether a true comorbidity exists (i.e., not confounded by sampling) it is important to estimate the prevalence of both isolated disorders and of comorbid cases from a large representative sample (Caron and Rutter, 1991). No large-scale study has investigated the prevalence of literacy, motor, and comorbid literacy and motor difficulties among a community-based sample. Although, as reviewed below, some small-scale studies of clinic- and community-based samples have been carried out.

Much of the work investigating the prevalence and profiles of comorbid dyslexia and DCD has used clinic-based samples (e.g., Kaplan et al., 2001; Dewey et al., 2002). One such early investigation by Kaplan et al. (1998) assessed motor, reading, and attention skills in 224 children referred for having learning or attention difficulties, along with 155 controls who had no reported difficulties. Despite no child being referred specifically for motor difficulties, the authors found 50% of the sample to meet their criteria for DCD. Using broad criteria for assessing reading ability (including both comprehension and word reading accuracy), 43.8% of the sample were identified as having dyslexia.

Of those who met the criteria for either DCD or dyslexia, 33% met the criteria for both disorders, suggesting one third of children presenting with either disorder had comorbid reading and motor difficulties. This high rate of comorbidity is somewhat surprising as no child was referred to the study for having motor problems. However, the rates reported in this study are likely inflated due to the recruitment of a clinic sample, and, due to the use of broad criteria for identifying disorders, particularly in reading disabilities (Caron and Rutter, 1991).

The prevalence of comorbid dyslexia and DCD has also been investigated in small community samples using parent/teacher questionnaires (e.g., Martin et al., 2010) or hybrid combinations of questionnaires followed up with behavioral assessments (Cruddace and Riddell, 2006). Cruddace and Riddell (2006) screened 129 children between 9 and 10 years of age using teacher reports of each child's reading and spelling, motor, and attention skills. Based on teacher identification, 68 children completed a behavioral battery and were categorized as having a reading and/or a motor difficulty. To establish prevalence estimates in their sample, the authors compared the number of children categorized as having dyslexia and/or DCD with the total number of children originally screened using teacher reports. Of the total sample, 21% of children met the criteria for reading difficulty, 23% for motor difficulty, and 13% for reading and motor difficulty. The frequency of reading and motor difficulties was below that reported by Kaplan et al. (1998) but more than double that would be expected based on the rates of the isolated disorders. Like Kaplan et al. (1998) and Cruddace and Riddell's (2006) data suggest an increased risk of comorbid literacy and motor difficulties.

Cruddace and Riddell (2006) report the prevalence estimates of comorbid reading and motor difficulties based on their sample probabilities and not population probabilities. However, the high incidence of isolated reading (11.6%) and motor (12.4%) difficulties in their sample was inflated in comparison to commonly reported population prevalence rates (e.g., Blank et al., 2012; Snowling, 2013). These very high rates of isolated difficulties may reflect the small sample size for a study of this nature and/or the use of teacher report questionnaires, which are not optimal for identifying reading and motor difficulties (Shaywitz et al., 1990; Blank et al., 2012; Barnett et al., 2015). The high base rates observed in the sample also raise questions about their validity. To conclude that there is an increased risk of comorbidity between dyslexia and DCD it is necessary to examine the frequency of comorbid difficulties in a sample where the rates of isolated dyslexia and DCD are similar to population prevalence estimates (Caron and Rutter, 1991). To date, no such study has been reported, although, Schoemaker et al. (2013) observed an increased risk of reading difficulties in a representative sample of children with motor difficulties who were part of a large community sample (the ALSPAC cohort; Lingam et al., 2009). Unfortunately, these authors did not examine the number of children with reading (and not motor) difficulties in the same sample, but nevertheless, these findings, along with those from Kaplan et al. (1998) and from Cruddace and Riddell (2006) suggest an above-chance risk of comorbidity between literacy and motor difficulties.

Building on the foregoing research, the aim of the first study reported here was to estimate the prevalence of isolated and comorbid literacy and motor difficulties in a representative community sample using a screening approach. Based on previous, smaller-scale studies, we expected to find the frequency of comorbid literacy and motor difficulties to be greater than expected based on the frequencies of children with isolated difficulties.

We used a community rather than a clinic sample, primarily to control for the aforementioned bias in clinic samples. This means that we did not recruit children who had a clinical diagnosis of dyslexia or DCD. Rather, we utilized types of measures that are often used to identify markers of these disorders (e.g., Blank et al., 2019). In the case of identifying DCD/motor difficulties we opted to measure handwriting and fine motor difficulties owing to the ease with which these skills can be estimated in large group settings, and because weaknesses in these skills are often the primary reason for referral for possible DCD (Miller et al., 2001).

It is also important to recognize that whilst screening tests, in the main, are useful for identifying those with likely difficulties in large samples, an individually administered assessment battery remains the most accurate assessment. To make the distinctions clear between (a) using a community rather than clinic sample and (b) screening vs. a more comprehensive assessment, we do not refer to our groups with the diagnostic terms *dyslexia* and *DCD*. Instead, we use the terms literacy difficulties and fine-motor difficulties for children who were categorized as having difficulties using the screening battery of Study 1. We use the terms literacy and motor disorders for children who we later identified in Study 2 to have significant markers of difficulties in the literacy and motor domains, on the basis of results from an individually administered diagnostic battery.

### Methods

#### Participants

To establish the prevalence of comorbid literacy and fine motor difficulties in a community-based (unselected) sample, 605 children took part in classroom screening. Children from six primary schools across North-West Wales participated in Years 3 (*n* = 204, *M*age = 8.2 years, *SD* = 0.52, 50% female), 4 (*n* = 200, *M*age = 9.1 years, *SD* = 0.54, 50% female), and 5 (*n* = 201, *M*age = 10.1 years, *SD* = 0.55, 48% female). We selected children in this age band, rather than younger children, to reduce the heterogeneity that is often seen in the profiles of younger children (aged < 7 years). This is in line with previous large-scale studies conducted in the U.K. examining children's literacy and motor skills (e.g., Lewis et al., 1994; Lingam et al., 2009). Prior to the start of data collection, 12% of children were identified by their schools as potentially having literacy difficulties, and, 3% of children were identified by their school as having diagnosed motor difficulties. All schools delivered instruction through the medium of English and the average proportion of pupils eligible for free school meals (a proxy of socioeconomic disadvantage) was 17%, in line with the national average for Wales (18%).

#### Procedure and Measures

Whole classes of children completed all tests in specially prepared booklets in a normal class setting. Any tests designed for individual administration were adapted for class administration; the adaptations are noted where relevant. Classes completed the booklets over two 60-min sessions to reduce fatigue effects. All groups received explanations and brief training prior to the beginning of each testing session to ensure they understood and complied with the instructions. The first author and two or three research assistants oversaw children's progress, along with the class teacher, to ensure good adherence to the test procedures. In the main, both sessions were completed on the same day with sessions separated with a break of at least an hour or within 1 week of each other. All performance scores for the measures comprising this screening, and their reliabilities are reported in Table 1.

**Table 1:** Descriptive statistics for measures of literacy and motor skills used in the screening battery as a function of school year group.

	**Year 3**		**Year 4**		**Year 5**		**Reliability**
	***M* (*SD*)**	**Range**	***M* (*SD*)**	**Range**	***M* (*SD*)**	**Range**	
**Literacy**
Word spelling							0.90^a^
Raw	24.73 (5.06)	6–41	27.77 (5.89)	12–48	30.24 (5.58)	17–44	
Standardized	105.68 (17.65)	56–144	105.65 (17.45)	56–145	105.09 (15.15)	67–145	
Sentence spelling	35.11 (11.51)	2–55	40.82 (11.27)	2–59	45.29 (9.71)	17–60	0.94^a^
Cloze reading	16.27 (5.02)	2–33	20.06 (5.73)	5–36	22.27 (6.96)	2–41	0.91^b^
**Fine motor**
VMI							0.73^a^
Raw	19.16 (2.59)	12–27	20.70 (3.07)	12–29	21.78 (3.50)	11–29	
Standardized	92.60 (9.98)	63–126	92.86 (12.16)	47–126	91.8 (13.87)	45–122	
Coding							0.85^c^
Raw	32.65 (8.05)	3–51	35.83 (8.25)	6–57	38.96 (8.30)	17–59	
Standardized	8.45 (3.05)	1–16	9.11 (2.70)	1–16	8.59 (2.54)	3–15	
Overall legibility	11.55 (2.12)	6.5–17.5	12.54 (2.5)	5.1–19.7	13.21 (2.73)	5.1–19.6	0.83^d^

*Standardized scores M = 100, SD = 15. VMI, Visual Motor Integration*.

a *Internal consistency (Cronbach's alpha) derived from the current, class administered, data*.

b *Test-retest correlation reported in Caravolas et al. (2005)*.

c *Average internal consistency reported in the WISC-IV manual (Wechsler, 2003)*.

d *Inter-rater (two-way random effects intra-class correlation)*.

##### Literacy Assessments

Word-level literacy measures were selected on the basis of ease of administration to classes. This meant that we could not use read-aloud measures of word reading (accuracy and fluency) that are typically administered to individuals. Evidence shows word reading and spelling tap the same word-level literacy construct and so we opted to administer word spelling tests (e.g., Kim et al., 2018). In addition, we used a cloze reading measure which relies on word reading accuracy as well as on broader comprehension (e.g., Keenan et al., 2008). As such, literacy skills were assessed using the Word Spelling subtest from WRAT-IV (Wide Range Achievement Test-IV; Wilkinson and Robertson, 2006), Sentence Dictation task from Caravolas et al. (2005), and the Cloze Reading task from Caravolas and Volín (2001).

*Word spelling.* The WRAT-IV Spelling test was adapted for classroom administration. In accordance with the manual, all participants first wrote 13 alphabet letters, after which, they were asked to write the first 36 words of this graded test. Each word was administered first in isolation, then within a carrier sentence, and a final time in isolation. We selected the first 36 words as the cutoff because this corresponds to a standardized score of 145 for a child in Year 5, and it was expected that most children would not surpass this score. Published guidelines were followed for administration and scoring. Each correct response received one point and scoring was discontinued after 10 consecutive errors.

*Sentence spelling.* We assessed spelling using a dictation task of 10 sentences. Sentence length varied from four to eight words. The sentences, comprising 62 words in total, were graded in their phonological, morphological, lexical, and orthographic complexity, in line with the national curriculum for England (c.f. Caravolas et al., 2005). Each correctly spelled word was awarded one point. This test was designed for group and individual administration.

*Cloze reading.* Children read short passages with missing words. For each missing word, they selected the most appropriate from a possible set of five word(s) printed below each passage. The first 14 passages were missing one word and the remaining 16 passages were missing two words. Passages varied between 7 and 45 words and were graded in complexity (c.f. Caravolas and Volín, 2001; Caravolas et al., 2005). Children read for 8 min, after which they were asked to stop and immediately put their pencils down. Each word correctly selected was awarded one point. This test was designed for group and individual administration.

##### Fine Motor Assessments

The lack of standardized motor assessments for the screening of motor skills in groups limited our choice of measures for use in the classroom. We therefore opted to use tests of perceptual-motor (e.g., visual motor integration) and handwriting skills. Whilst perceptual-motor skills are utilized in all types of skilled motor action (Halsband and Lange, 2006), the tasks used in this study are arguably those most related to fine-motor skills and less so to other motor functions such as gross motor skills or balance. We used several measures of fine motor skills including the Beery Visual Motor Integration Test-VI (VMI-VI; Beery and Beery, 2010), the Coding subtest from the Wechsler Intelligence Scale-IV (WISC-IV; Wechsler, 2003), and Handwriting Legibility scores from the Spelling and Handwriting Legibility Test (SaHLT; Caravolas et al., in preparation). We used handwriting skills as a marker for fine-motor difficulties because poor handwriting and fine motor problems, are predominant reasons for referal of children with DCD (Miller et al., 2001); morevoer, poor performance on handwriting measures has been found to descriminate well between children with and without DCD (Rosenblum and Livneh-Zirinski, 2008; Rosenblum et al., 2013). Therefore, the inclusion of fine-motor and handwriting skills in a screening battery was likely to result in reasonably good sensivity of a screening battery to detect broader motor problems.

*Visual motor integration.* This was assessed by the Beery VMI-VI (Beery and Beery, 2010). Children copied a series of 24 shapes of increasing complexity. They copied the shapes exactly as the saw them into a box directly below each item without using any additional aids (rulers, rubbers, etc.). Only one attempt was allowed per item. Scoring followed published guidelines and each correctly copied item was awarded one point. Scoring was discontinued after three consecutive items were awarded one point.

*Coding.* Coding tests place demands on visual-motor speed and accuracy (Sattler, 2001) and have been used as a proxy of graphomotor speed previously (Caravolas et al., 2001; c.f. Sattler, 2001) and we used the WISC IV Coding subtest (Wechsler, 2003) for the same purpose here. In an adaptation for group administration, children used a numbered key of symbols printed at the top of the page to reproduce the corresponding symbol into a numbered box located in the second half of the page as quickly as possible in 2 min. Scoring followed published guidelines and responses were scored as correct if they were identifiable as the relevant symbol.

*Handwriting legibility.* We assessed handwriting legibility using the protocol from the SaHLT (Caravolas et al., in preparation; see also Caravolas et al., 2020). Children's handwritten responses to the sentence dictation task were scored on four dimensions as follows: (a) Letter Formation, which measures the child's accuracy and consistency in producing letters; (b) Letter Spacing, which assesses the child's ability to appropriately and consistently space letters within words; (c) Word Spacing, which evaluates the child's ability to appropriately and consistently spaces words within a sentence; (d) Line Alignment, which gauges the degree to which the child can write along the line. Each of the four dimensions was scored using a 5-point Likert scale ranging from 1 *highly illegible* to 5 *highly legible*. The dimensions were applied to each sentence individually. The scores for each dimension were calculated by averaging the score across the number of sentences the child wrote. An Overall Legibility score was derived by summing the average dimension scores. This test was designed for group and individual administration.

#### Ethics

Both studies were approved by the School of Psychology's Research Ethics Committee at Bangor University (reference: 2015-15287) and an NHS Research Ethics Committee (reference: 16/WA/0141). They were conducted in accordance with the British Psychological Society Code of Ethics and Conduct.

### Results

#### Descriptives

The means, standard deviations, ranges, and reliabilities for the measures administered in the screening battery are reported in Table 1. The descriptive statistics on the raw scores show large variations in ability across all measures without evidence of floor or ceiling effects. Increases in performance with increasing school years is apparent for all measures. Reliability estimates suggest good-to-excellent reliability for all measures. We also report norm-referenced standardized scores, where available, to assess whether the group-administration produced any aberrant patterns of performance relative to the performance patterns obtained from individual administration. Note, importantly, that we did not use the published standard scores reported in Table 1 in our further statistical analyses. For the latter purpose, we computed internal standard scores (z-scores) from the raw scores obtained in the present study. The means, standard deviations, and ranges of Word Spelling standardized scores also show a large variation in ability, with averages in the normal range reflecting the unselected sampling method we used. Performance on the fine-motor tasks (VMI and Coding) was on average lower than Word Spelling, however, average performance was still within the normal range. It is also important to note that the reliability of the VMI was relatively lower than all the other tests. However, the reliability derived from the current sample is not too dissimilar from the published reliabilities for children in these year groups (α = 0.79–0.81; Beery and Beery, 2010). To investigate potential subclinical motor difficulties in children with literacy difficulties we plotted the score distributions for each group on each measure (see Supplementary Figure 1). We found large variations in groups, but that the distribution of children with LD was fully overlapping with that of the typically developing group, suggesting the absence of subclinical motor difficulties in this group.

#### Prevalence Estimates

To assess the prevalence of literacy and fine-motor difficulties separately, and in co-occurrence in individual children, we used a marker approach (see Snowling and Hulme, 2015). Often studies examining literacy or motor disorders apply diagnostic cut-offs of between 1 and 1.5 *SD* below the age or year group average (e.g., Lewis et al., 1994; Blank et al., 2019). In deciding cut-offs for this study, we followed the recommendation of Rutter et al. (2004) to strike a balance between identifying children who have clear difficulties while ensuring a sufficient number of children to obtain representative and accurate base rates. Therefore, we decided 1.33 *SD* was an appropriate cut-off. To apply this, we generated z-scores (*M* = 0, *SD* = 1) as a function of year group on a selection of the literacy and motor tests administered. Thus, children who scored below the cut-off of < 1.33 *SD* of their year group average on two out of three of the selected literacy tests—Word Spelling, Sentence Spelling, or Cloze Reading—were identified as having potential literacy difficulties. Children who scored below the cut-off on two out of the three selected fine-motor measures—Visual Motor Integration, Coding, and Total Handwriting Legibility—were identified as having potential fine-motor difficulties. Children who met the criteria for both literacy and fine-motor difficulties were identified as having potential comorbid literacy and fine motor difficulties. Children who did not meet any criteria were labeled as typically developing (TD).

The prevalence estimates of literacy, fine-motor, and comorbid literacy and fine-motor difficulties are reported in Table 2. These isolated disorder prevalence rates are broadly in line with previous epidemiological studies of dyslexia and DCD, respectively (Lingam et al., 2009; Snowling and Hulme, 2015). To determine whether the frequency of comorbid literacy and motor difficulties exceeded that expected by chance, the derived base rates of isolated literacy and motor difficulties were multiplied to obtain the percentage of expected cases of comorbid difficulties. Following these procedures described by Caron and Rutter (1991) and Landerl and Moll (2010), the expected rate (*n* = 3, 0.54%) was then compared to the number of observed cases (*n* = 16, 2.64%) meeting our criteria for comorbidity. The observed frequency of children with comorbid literacy and fine-motor difficulties was significantly higher than that expected by chance (OR = 5.78, *p* < 0.001), suggesting that comorbid literacy and motor difficulties cannot be attributed to chance alone.

**Table 2:** Proportion of children in the sample identified as having literacy, fine motor, comorbid difficulties, or as being typically developing.

	***n***	**%**
Literacy difficulties	42	6.94
Fine-motor difficulties	34	5.62
Comorbid literacy and motor difficulties	16	2.64
Typically developing	513	84.79

In sum, we found the measures in the screening battery to be reliable in assessing literacy and fine-motor skills. Furthermore, the rates of isolated literacy and fine-motor difficulties derived from this battery were in line with previous studies examining the prevalence of these difficulties in British children (Lingam et al., 2009; Snowling and Hulme, 2015). Such plausible base rates are critical for determining whether the rate of comorbidity between literacy and (fine-)motor difficulties exceeds chance significantly. Indeed, the rate of comorbid literacy and fine-motor difficulties was five times greater than expected by chance. In what follows, we extended the current findings in a second study by (a) assessing the sensitivity and specificity on the screening measures, and (b) examining the cognitive profiles of children with literacy, motor, and comorbid literacy and motor disorders.

## Study 2

The greater-than-chance incidence of comorbid literacy and motor difficulties reported in Study 1 presents tentative support for the claim that these disorders are to some extent related. Pennington's (2006) multiple deficit model (MDM) explains this relationship in the context of shared etiological and cognitive risk factors, where each disorder results from numerous biological and cognitive risk factors that act in a probabilistic manner to increase the likelihood of an individual meeting a diagnostic threshold. Some of these risk factors are specific to a disorder, that is, they are independent, whilst others are shared between disorders. The presence of shared risk factors increases the likelihood of comorbidity between the disorders. This hypothesis has led to a proliferation of studies investigating independent and shared risk factors of dyslexia (e.g., Gooch et al., 2011; Moll et al., 2016). To date, however, it remains unclear what are the independent and shared risk factors of literacy and motor disorders.

Studies investigating each of these disorders separately suggest that some cognitive deficits are observed in both. In Study 2, we investigated the reported co-incidence of deficits in phonological processing, visuospatial processing, memory, and selective attention. Below, we briefly evaluate the literature reporting the potential overlap of deficits in the cognitive domains of literacy and motor disorders.

Variations in phonological skills are a critical determinant in learning to read and spell (Caravolas et al., 2012; Melby-Lervåg et al., 2012). Children with dyslexia typically experience phonological processing deficits (e.g., Snowling, 2008), which precede and predict their later literacy (dis)abilities (Pennington and Lefly, 2001; Hulme et al., 2015). Moreover, effective training in phonological skills improves the phonological and literacy skills of children at risk of or experiencing dyslexia (e.g., Hulme et al., 2012). Thus, phonological deficits are common in dyslexia and are causally related to the disorder.

Notably, however, some children who have phonological deficits go on to develop typical reading and spelling skills, while others with poor literacy do not appear to have phonological deficits (Ramus et al., 2003). Therefore, phonological deficits by themselves may not be sufficient to cause dyslexia/literacy difficulties. Rather, phonological deficits act probabilistically (rather than deterministically) with other cognitive deficits to increase the risk for a child to meet diagnostic criteria for dyslexia (Pennington et al., 2012; Moll et al., 2016).

Difficulties on measures, which require phonological skills, have also been reported amongst some children with motor disorders. Case-control studies report that children with DCD perform less well than children without DCD on measures such as non-word reading and repetition, as well as on word reading and spelling (Alloway, 2007; Archibald and Alloway, 2008; Schoemaker et al., 2013). However, the reported prevalence of weaknesses in phonological and literacy skills among children with DCD is highly variable.

There are several potential explanations as to why children with motor difficulties may struggle on phonological, reading, and spelling tasks. One possibility is that phonological and literacy difficulties are a distal consequence of motor deficits. For example, oral-motor or graphomotor deficits may interfere with learning to read and write. Another potential, but unexplored, explanation for phonological deficits amongst children with motor difficulties could be the presence of children with comorbid literacy difficulties in the samples studied. For example, despite the variability observed in their sample, Dewey et al. (2002) did not discriminate between children with phonological deficits who had literacy impairments (i.e., those with comorbid dyslexia) and those who did not. Finally, visuospatial skills have been reported to also be involved in reading acquisition, in addition to phonological skills (Franceschini et al., 2012), and, children with motor difficulties often have visuospatial deficits (see below). In line with this view, some studies have found that children with motor difficulties struggle on reading tasks due to visuospatial deficits (Bellocchi et al., 2017).

Visuospatial skills are functional in localizing visual information and providing feedback for correction of goal directed movements (e.g., Wolpert and Ghahramani, 2000), hence they are important for acquiring and making skilled motor actions (Halsband and Lange, 2006; Jeannerod, 2006). It is not surprising, then, that children with motor difficulties tend to perform poorly on visuospatial tasks regardless of whether they require a motoric response. They are also reported to be impaired on tasks of visual perception without a motor component (Hulme et al., 1982; Tsai et al., 2008) and on visual-motor integration (Schoemaker et al., 2001; Bonifacci, 2004). Meta-analyses have confirmed large differences between children with and without DCD on tasks involving visuospatial processing (Wilson and McKenzie, 1998; Wilson et al., 2013).

Despite moderate-to-large group effects on these tasks, the relationship between visuospatial processing and motor disorders is unclear. Whilst some have found significant correlations between visuospatial processing and functional motor skills in children with DCD (Lord and Hulme, 1987; Tsai and Wu, 2008) others have reported no associations (Prunty et al., 2016). Such mixed findings and lack of longitudinal and training investigations examining the relationships between these abilities preclude strong claims about the causal role of visuospatial processing deficits in motor disorders. Nevertheless, the strong association between visuospatial skills and typical motor development as well as the clear difficulties of children with DCD on tasks involving visuospatial processing suggest that poor performance on visuospatial tasks is a probable cognitive risk factor of motor disorders.

Impairments in visuospatial and motor skills have been reported amongst children with literacy disorders (Ramus et al., 2003; Bellocchi et al., 2017). Yet, few studies have fully controlled for a comorbid motor disorder. In one study that did control for comorbidity, children with dyslexia scored within the average range on measures of visual perception and visual-motor integration, and better than children with isolated and comorbid DCD (Bellocchi et al., 2017). Thus, children with a comorbid motor disorder may have accounted for the visuospatial processing impairments reported in this study, however, the lack of a typical control group made it difficult to rule out the presence of a sub-clinical visuospatial deficit in dyslexia.

Children with a literacy disorder are known to perform less well than typically developing children on various memory measures—verbal memory being the most strongly affected (Kudo et al., 2015)–, albeit their verbal memory impairments tend to be smaller than their phonological deficits (Melby-Lervåg et al., 2012). In children with motor difficulties, memory impairments appear to be more diffuse with greater severity in the visual memory domain (Blank et al., 2012). More recently, a study by Maziero et al. (2020) directly compared performance on memory measures between children with dyslexia and DCD. The authors reported dyslexia was most strongly associated with verbal memory deficits whereas DCD was most strongly associated with visual memory deficits. However, measures of visual memory tap heavily on visuospatial processing, which is itself a skill often impaired in motor disorders. This potential confound is yet to be disambiguated.

Like memory, attention is not a unitary construct. According to one view, attention is divided into three sub-processes, namely sustained, selective, and control (Shapiro et al., 1998; Manly et al., 2001). In the present article, we focus on selective attention, that is, the enhanced capacity of processing specific stimuli. Impairments on measures of selective attention have been reported in children with literacy disorders (Menghini et al., 2010; Varvara et al., 2014) and motor disorders (Wilson et al., 1997). Cruddace and Riddell (2006) reported that groups with dyslexia, DCD, and comorbid dyslexia and DCD all attained relatively low scores on selective attention measures, as did the control group, and no statistical differences were observed between groups. Thus, either the measure under study was not sufficiently sensitive to detect selective attention deficits in the disorder groups, or, such deficits do not characterize these groups.

In establishing whether deficits in memory and selective attention are present in literacy and/or motor disorders, it is necessary to rule out potential confounds such as uncontrolled comorbidity and measurement issues. It is also important to note that memory and attention deficits are unlikely to be direct causes of literacy or motor disorders. Rather, deficits in these domain-general skills are more likely to interact with disorder-specific deficits to compound impairments and increase the likelihood of a child meeting a diagnostic threshold (Gathercole et al., 2016).

The above studies investigating phonological, visuospatial, memory, and selective attention deficits in dyslexia and DCD have predominantly examined their presence in one or the other disorder, but not both. Despite the seemingly high degree of overlap in cognitive deficits across studies, it is unclear in the vast majority of cases whether researchers have controlled for the potential comorbidity between the disorders. It is therefore timely to examine the incidence of deficits in these four cognitive domains among groups with isolated or comorbid literacy and motor difficulties. In doing so, we will delineate their cognitive profiles and allow the identification of independent and shared deficits between the disorders.

Beyond identifying shared and independent risk factors for literacy and motor disorders, it is also important to examine how children with comorbidity in these domains perform relative to children with isolated disorders. Such comparisons allow us to test competing explanations of comorbidity. Three main competing explanations of the etiology of comorbid developmental disorders have been postulated (de Jong et al., 2006). The phenocopy hypothesis suggests that a single etiology underlies the cognitive deficits consistent with an isolated disorder, but these deficits lead to behavioral manifestations of a second disorder (Pennington et al., 1993). In this view, children with comorbid literacy and motor disorders would have cognitive deficits that were only consistent with one or the other disorder. Alternatively, the cognitive subtype hypothesis suggests the comorbid disorder is a third disorder with a separate etiology to either of the isolated disorders (Rucklidge and Tannock, 2002). According to this hypothesis, children with comorbid literacy and motor disorders would have a different profile and/or greater severity of deficits to either isolated literacy or motor disorder. Finally, the shared etiology hypothesis suggests that comorbid disorders share at least some common etiology. Accordingly, children with comorbid literacy and motor disorders would have a similar profile of deficits—not differing in severity—to isolated literacy or motor disorders.

The phenocopy and cognitive subtype hypotheses follow a single deficit account while the shared etiology hypothesis is consistent with the MDM, which posits that comorbidity results from shared etiological and cognitive risk factors (Pennington, 2006). Recent studies examining the comorbidities between dyslexia and ADHD (Gooch et al., 2011) and reading and math disorder (Moll et al., 2016) find support for the latter view. However, to date, no study has comprehensively examined whether this account holds true for comorbid literacy and motor disorders. A study by Biotteau et al. (2017a) that examined general cognitive and attention abilities in children with dyslexia, DCD, and comorbid dyslexia and DCD found no differences between the comorbid and isolated disorder groups. These findings are most consistent with the shared etiology hypothesis. In line with this evidence, we predict that the profiles of comorbidity between children with literacy and motor disorder will follow that of the shared etiology account.

To investigate potential shared and independent risk factors for literacy and motor disorders and to delineate the nature of comorbid literacy and motor disorders, we invited children who we identified as having difficulties or being typically developing in Study 1 to complete a comprehensive battery of individually administered tests. Each child underwent a battery of 16 tests to assess functioning in the domains of literacy, fine and gross motor abilities, phonological processing, visuospatial processing, memory, and selective attention. This comprehensive assessment with a subsample of children from the previous study allowed us to validate the sensitivity and specificity of the screening battery (Study 2a), and to increase the accuracy of each child's group classification. Furthermore, children's scores on the tests of the broader battery were analyzed to elucidate the cognitive profiles of each disorder group (Study 2b).

## Study 2A: Validity of the Screening Test Battery

Prior to examining profiles of cognitive deficits in literacy and motor disorders, we first examined the validity of the screening assessments used in Study 1 in identifying children with literacy and/or motor difficulties. In particular, we assessed the screening battery's ability to correctly categorize children with a significant difficulty (sensitivity) and those without significant difficulty (specificity). To this end, we carried out a discriminant function analysis where all children in Study 2a were assigned to a group of literacy disorder, motor disorder, co-occurring literacy and motor disorders, or typical development, independently of their group classification in Study 1, but rather on the basis of their performance on new individual assessments of literacy and motor skills (see details below). The discriminant function analysis was used to determine the sensitivity and specificity of classification to a disorder group by the screening battery relative to the group membership as determined by the results of more extensive battery of Study 2a.

### Methods

#### Participants

A total of 153 children from Study 1 and their parents consented to take part in this second study. Children were now in Years 4 (*n* = 47, 51% female; *M*age = 105.93 months, *SD* = 3.76), 5 (*n* = 53, 40% female; *M*age = 117.62 months, *SD* = 4.42), and 6 (*n* = 53, 43% female; *M*age = 130.36 months, *SD* = 4.84). No child was reported to have received a new diagnosis of literacy and/or motor disorder between Study 1 and Study 2.

#### Procedure and Measures

Approximately 4 months after Study 1, children completed a large battery of the diagnostic measures described in Study 2a and 2b. The battery, administered over 5 individual sessions, included multiple measures of literacy and motor skills, phonological, visuospatial processing, memory, and attention skills. Within each testing session, the administration order of the individual tests was fixed and manipulated to minimize the likelihood of transfer, or priming, from one test to the other. Each testing session lasted no longer than 1 h, and children were given an opportunity to take a short break after each test. Published administration and scoring instructions, including any discontinuation criteria, were followed.

##### Literacy Skills

This was assessed by three reading tests: WRAT-IV Single Word Reading subtest, 1 Min Word Reading Test and 1 Min Pseudoword Reading tests from the Multilanguage Assessment Battery of Early Literacy (MABEL; Caravolas et al., 2019).

*Word reading accuracy.* The Single Word Reading subtest was used to assess reading accuracy. The child was asked to read aloud from a 55-item graded word list. Words increased in difficulty and administration of basal and ceiling levels was carried out according to published guidelines. Each correctly read item was awarded one point. Internal reliability was α = 0.94.

*One-minute word reading.* The child was asked to read aloud as many words as s/he could from a list of 144 high frequency words in 60 s. The words increased in length (one to eight letters) and in syllable number (one to three syllables). Each correctly read word in the time limit was awarded one-point. Reported test-retest reliability was *r* = 0.91 (Caravolas, 2017).

*One-minute pseudoword reading.* Following the same procedure as the 1 Min Word Reading, the child read aloud from a list of 144 pseudowords as fast as they could in 1 min. Each pseudoword read plausibly in the time limit was awarded one-point. Reported test-retest reliability was *r* = 0.87 (Caravolas, 2017).

##### Motor Skills

Motor ability was measured using the Beery VMI-VI Motor Coordination subtest (Beery and Beery, 2010), Lace Threading and One Board Balance from the Motor Assessment Battery for Children 2 (MABC-2; Henderson et al., 2007).

*Motor coordination.* The child traced as accurately as possible inside 24 shapes of increasing complexity. Only one attempt was allowed per form and children were asked to stop after 5-min, although most children completed the task in this time. Scoring followed detailed guidelines reported in the manual. Each correct response was awarded one point. Scoring was discontinued when a child made three consecutive errors. Internal reliability was α = 0.70.

*Lace threading.* The child threaded a piece of string back and forth through eight holes in a small plastic board. The task was timed from when the child's hands—positioned on the table either side of the board—left the mat, until they had pulled the string tight through the final hole. The threading time was the fastest time of two consecutive attempts. The intraclass correlation (ICC) was 0.61.

*Balance board.* Static balance was measured by the One Board Balance test where the child balanced with one foot on a plastic board with a thin keel. Once the child had achieved a balanced position, the administrator began timing and continued for up to 30 s or when balance was lost. Two attempts of balancing for up to 30 s were allowed per foot. The ICC was 0.64 for the right foot and 0.62 for the left foot.

### Results

The descriptive statistics of the measures across all participants are reported in Supplementary Table 1. As expected, there was wide variation in ability but there was no indication of floor or ceiling effects in any of the measures. All measures' reliability ranged from acceptable-to-excellent.

#### Group Classification

Performance on the literacy and motor assessment battery was used to identify whether each participant had literacy, motor, or comorbid literacy and motor disorders, or was typically developing. We used a similar approach to Study 1 and a child was identified as having a *literacy disorder* if they scored 1.33 *SD* below their age average on two out of the three literacy tests. They were identified as having a *motor disorder* if they performed 1.33 *SD* below their age average on two out of the three motor tests. If they met the criteria for both literacy and motor disorder, children were identified as having *comorbid literacy and motor disorder*. Those who did not meet any criteria were classified as being *typically developing*.

The characteristics of each of the four groups, along with the statistical comparisons of the groups on classification measures, are reported in Table 3. Groups did not differ in age but children with comorbid disorders had significantly lower non-verbal ability. Children with literacy disorder only had significantly lower scores on all literacy measures, as expected, but did not differ from typically developing children on motor measures. Children with motor disorder had significantly lower scores on all motor measures, as expected, but did not differ from typically developing children on literacy measures. Children classified as having comorbid literacy and motor disorder achieved significantly lower scores than typically developing children on all literacy and motor tests.

**Table 3:** Group demographics and performance on classification measures.

	**LD**		**MD**		**LD+MD**		**TD**		**Group comparison**	
	***M***	***SD***	***M***	***SD***	***M***	***SD***	***M***	***SD***	***F***	**$\eta_{\rho }^{2}$**
*n*	27		24		17		85		–	–
% Female	33		29		59		49		–	–
Age (months)	118.26	10.54	118.5	10.36	118.35	13.24	118.38	10.92	0.00	<0.01
Block design (NVIQ)^a^	9.88	3.70	8.73	2.76	6.53^**^	2.56	9.91	3.29	5.11^**^	0.10
**Literacy**^**†**^
Single word reading^c^	82.26^***^	8.51	101.00	11.50	80.82^***^	6.95	101.82	9.93	44.38^***^	0.47
One minute word read^b^	66.42^***^	14.14	87.76	13.40	70.47^***^	20.19	96.48	13.90	34.20^***^	0.43
One minute pseudoword read^b^	25.22^***^	12.46	43.71	12.23	21.71^***^	12.14	48.59	13.80	34.81^***^	0.41
**Motor**^**‡**^
Motor coordination^c^	91.11	7.18	75.54^***^	9.08	76.88^***^	8.22	91.78	9.44	30.53^***^	0.38
Threading^a^	8.81	2.37	6.64^***^	3.20	5.33^***^	2.23	9.64	2.78	14.78^***^	0.24
One board balance^a^	9.92	2.37	8.78^**^	3.37	7.59^***^	2.62	10.78	2.37	9.23^***^	0.16

*LD, literacy disorder; MD, motor disorder; LD+MD, comorbid literacy and motor disorder; TD, typical developing*.

† *Measures used to classify literacy disorder*.

‡ *Measures used to classify motor disorder. Subscript asterisks of group mean represent significant differences from Bonferroni corrected post-hoc comparisons with typically developing children*.

a Scaled scores;

b *Raw scores*,

c *Standardized scores*.

** *p < 0.01*,

*** *p < 0.001*.

#### Validity of the Screening Battery

Predictive discriminant function analysis (DFA) was run to assess whether tests of literacy and motor-related skills administered in Study 1 predicted group membership in Study 2a. Accordingly, performance on the tests in Study 1 were entered as predictors for the classification of literacy or motor disorder, and typically developing children in Study 2a. Predictive DFA requires groups classified to be mutually exclusive and so we did not attempt to classify comorbid literacy and motor disorders here. Given the unequal sample sizes, we also set group-size-proportional prior probabilities. The top section of Table 4 shows the canonical correlations for function 1, χ^2^ = 0.36, *F*_(12, 222)_ = 12.39, *p* < 0.001, and function 2, χ^2^ = 0.74, *F*_(5, 112)_ = 7.78, *p* < 0.001. Both functions are statistically significant indicating they are both needed to describe differences between the classifications.

**Table 4:** Discriminant function analysis of Study 1 measures in classifying literacy and motor disorders showing canonical correlations (top section), loadings (mid-section), and group means (bottom section) of each function.

	**Function one**	**Function two**
**Canonical correlations**
	0.72^***^	0.51^***^
**Canonical loadings**
Sentence spelling	0.92	0.23
Word spelling	0.70	0.14
Cloze reading	0.62	−0.07
Visual motor integration	0.10	0.68
Coding	0.18	0.77
Overall legibility	0.07	0.85
**Group means on canonical variables**
Literacy difficulties	−1.92	0.13
Motor difficulties	0.30	−1.15
Typically developing	0.62	0.35

*** *p < 0.001*.

The canonical structure (mid-section of Table 4) reveals high loadings for literacy measures on function one and high loadings for motor-related measures on function two. Furthermore, children later classified as having a literacy disorder had the lowest group mean on function one whereas children with motor disorder had the lowest group mean on function two (bottom section of Table 4). The battery achieved sensitivity and specificity rates of 86 and 95%, respectively, for identifying literacy difficulties which exceeded the recommended limits of 80 and 90% for sensitivity and specificity rate, respectively, for screening tools of this nature (Glascoe and Byrne, 1993). The battery also achieved sensitivity and specificity rates of 79 and 84% respectively for identifying motor disorder. These rates are recognized as being “good” for motor assessments (see Blank et al., 2019). Therefore, the literacy and fine-motor screening assessments used in Study 1 had relatively good sensitivity and specificity in detecting literacy and motor disorders, assessed using a broader individually administered battery.

## Study 2B: Cognitive Profiles

Having established that comorbidity between literacy and motor difficulties is greater than chance (Study 1) and having validated the aforementioned screening battery (Study 2a), we sought to examine group profiles across the four cognitive domains of phonological processing, visuospatial processing, memory, and attention. By examining profiles across children with literacy, motor, and comorbid literacy and motor disorders, we sought to elucidate independent and shared risk factors for these disorders, as well as to identify whether comorbid literacy and motor disorder is most consistent with the phenocopy, cognitive subtype, or shared etiology hypothesis.

### Method

#### Procedure and Measures

Tests of visuospatial processing, phonological processing, memory, and attention were administered as part of the same battery of tests measuring literacy and motor skills reported in Study 2a.

##### Visuospatial Processing

Both motor and non-motor visuospatial processing were measured using scores from the Beery VMI (described in Study 1; Beery and Beery, 2010) and Visual Perception subtest (Beery and Beery, 2010). We also derived a third measure of visuospatial processing from the Wide Range Intelligence Test (WRIT; Glutting et al., 2000).

*Visual perception.* The child was asked to look carefully at a shape (target) printed at the top of the page and select and mark the shape that matched the target from several distractor shapes as accurately as possible. The complexity of the shapes increased as did the number of distractor shapes from two to seven. Each shape correctly identified in 3 min was awarded one-point until the child met the discontinue rule of three consecutive errors. The reported reliability of this measure was acceptable (α = 0.71).

*Matrix visual perception.* Matrix reasoning tests tap, in part, visuospatial processing (Sattler, 2001; Stephenson and Halpern, 2013). As such, the first author along with a research assistant each selected items from the WRIT Matrix Reasoning subtest (Glutting et al., 2000), which fulfill the criteria of visuospatial processing as outlined in the Test of Visual Perceptual Skills-4 (TVPS-4; Gardner, 2017). Inter-rater reliability was excellent between the first author and the research assistant (Kappa = 0.84) and internal reliability of the selected items was acceptable (α = 0.73).

##### Phonological Processing

Phonological processing was measured using the Phoneme Deletion, Phoneme Blending, and Rapid Automatized Naming (RAN) tasks from the MABEL (Caravolas et al., 2019).

*Phoneme deletion.* The child was asked to repeat a pseudoword after removing either the initial (10 items, onsets) or final (10 items, codas) phoneme. Performance was measured in term of accuracy, which was expected to be high in these age groups, and in terms of speed. As such, speed was used as the measure of performance. The intraclass correlation (ICC) for the speed measure was 0.86 for onsets and 0.76 for codas.

*Phoneme blending.* The child was asked to synthesize speech sounds (phonemes) presented at 1 s intervals into real words. The test comprised 24 target words of increasing length and phonological complexity. Internal reliability was α = 0.78.

*Rapid automatized naming (RAN).* Two variants of the RAN task—*digits and letters*—were administered. In each case, the child named the stimuli presented on two A4 display cards, with 8 items repeated pseudo-randomly in two arrays of eight by five from left to right. During the RAN Digits subtest the child was asked to name the digits: 2, 3, 6, 7, and 9. In the RAN Letters subtest, the child was asked to name the lowercase letters: a, d, p, o, and s. Accuracy is usually at ceiling in RAN tasks and so we used speed as the measure of performance. The ICC for the speed measure was 0.95 for RAN Digits and 0.92 for RAN Letters. Furthermore, we produced a composite of RAN by averaging the scores of both variants.

##### Memory

Verbal memory was measured using the Digit Span task from the WISC-IV (Wechsler, 2003) and visual memory using the Block Recall task of the Working Memory Test Battery for Children (WMTB-C; Gathercole and Pickering, 2001).

*Verbal memory.* Both forward and backward digit span was measured. In the forward subtest, the child was asked to recall sequences of single digit numbers the administrator read aloud. In the backward subtest, the child was asked to recall the single digit numbers in the reverse order. The sequence length increased from two to nine digits and the child recalled two trials per sequence length. Administration was discontinued when the child was unable to recall two trials of the same string length. The reported internal reliabilities for forward was α = 0.83 and backward was α = 0.80.

*Visual memory.* The child tapped the same sequence of blocks as was demonstrated by the administrator. The span of blocks in the sequence increased from one to nine. Each span had a total of six trials and one point was awarded per correct trial. The test was stopped when the child made three errors in one span. The reported reliability was α = 0.76.

##### Selective Attention

Selective attention was measured using subtests from the Test of Everyday Attention for Children (TEA-Ch; Manly et al., 2001).

*Sky search.* The child was asked to circle pairs of spaceships comprising the same design (target) hidden amongst other pairs of spaceships composed of different designs (distractors) under speeded conditions. Two measures were derived from this task. *Time per target* was the time taken to complete task divided by the number of correctly circled target pairs. The *attention score* was the time per target minus the time per target of a motor control block (circle only visible targets). Reported test-retest reliability was *r* = 0.90.

*Sky search DT.* The child was asked to complete Sky Search again (using stimuli presented in a different order) whilst s/he counted sounds played via tape. Here, we used time per target (time taken to complete task divided by the number of correctly circled target pairs) as the measure of attention. Reported test-retest reliability was *r* = 0.81.

### Results

We sought to (a) identify shared and independent cognitive risk factors of literacy and motor disorders and (b) to examine the profiles of children with isolated disorders relative to children with comorbid disorders to elucidate the nature of literacy and motor disorders comorbidity. Thus, we compared groups of measures of visuospatial processing, phonological processing, memory, and selective attention (see Supplementary Table 2 for descriptive statistics of individual measures). As multiple measures of the same constructs were administered and there was a large variation in age, we used a Multiple Indicators Multiple Cause (MIMIC) model, regressing age, to confirm the validity of the battery and to derive factor scores for group comparisons.

#### Correlations

Pearson correlations between all measures (raw scores) aggregated across the whole sample (reported in Table 5) were conducted to examine the relationships between measures of the same and different constructs. There were significant correlations between age and all other variables, with the exception of the memory measures, indicating that attainment increased as children got older. On the whole, though, measures of the same construct had the highest intercorrelations, indicating convergent validity.

**Table 5:** Pearson correlations among measures of visuospatial processing, phonological processing, memory, and selective attention skills.

	**1**	**2**	**3**	**4**	**5**	**6**	**7**	**8**	**9**	**10**	**11**	**12**
1. Age (months)
Visuospatial processing
2. Visual perception	0.21^**^											
3. Matrix visual perception	0.19^*^	0.24^**^										
4. Visual motor integration	0.11	0.43^***^	0.21^**^									
Phonological processing
5. Phoneme deletion	−0.25^**^	−0.21^**^	−0.23^**^	−0.26^***^								
6. RAN	−0.25^**^	−0.16	−0.16^*^	−0.11	0.60^***^							
7. Phoneme blending	0.22^**^	0.24^**^	0.20^*^	0.19^*^	−0.21^**^	−0.32^***^						
Memory
8. Forward verbal span	0.12	0.33^***^	0.14	0.31^***^	−0.20^*^	−0.19^*^	0.29^***^					
9. Backward verbal span	0.13	0.19^*^	0.02	0.23^**^	−0.32^***^	−0.32^***^	0.19^*^	0.35^***^				
10. Visual span	0.08	0.33^***^	0.10	0.36^***^	−0.31^***^	−0.28^***^	0.23^**^	0.25^**^	0.40^***^			
Selective attention
11. Sky search	−0.26^***^	−0.21^*^	−0.20^*^	−0.19^*^	0.44^***^	0.46^***^	−0.20^*^	−0.14	−0.31^***^	−0.25^**^		
12. Sky search TPT	−0.35^***^	−0.22^**^	−0.22^**^	−0.25^**^	0.46^***^	0.48^***^	0.21^**^	−0.14	−0.31^***^	−0.28^***^	0.90^***^	
13. Sky search DT TPT	−0.26^***^	−0.21^*^	−0.23^**^	−0.35^***^	0.35^***^	0.33^***^	−0.13	−0.18^*^	−0.31^***^	−0.26^***^	0.52^***^	0.65^***^

*TPT, time per target*.

*** *p < 0.001*,

** *< 0.01*,

* *p < 0.05*.

#### Factor Analyses

Analyses were run using full information maximum likelihood estimation in Mplus 7.2 (Muthén and Muthén, 2014) due to the small amount of missing data (< 2% across all measures). A four-factor (visuospatial processing, phonological processing, selective attention, and memory) baseline confirmatory factor analysis (CFA) was run where all indicators loaded onto their respective factors. Phoneme Blending and Forward Digit Span were correlated as were Sky Search and Sky Search DT to account for the variance shared by similar task demands. The final baseline model produced an acceptable fit, χ^2^(46) = 57.20, *p* = 0.125, RMSEA = 0.040 [90% CI = 0.000, 0.070], SRMR = 0.061, CFI = 0.98, and TLI = 0.98.

**Figure 1:**
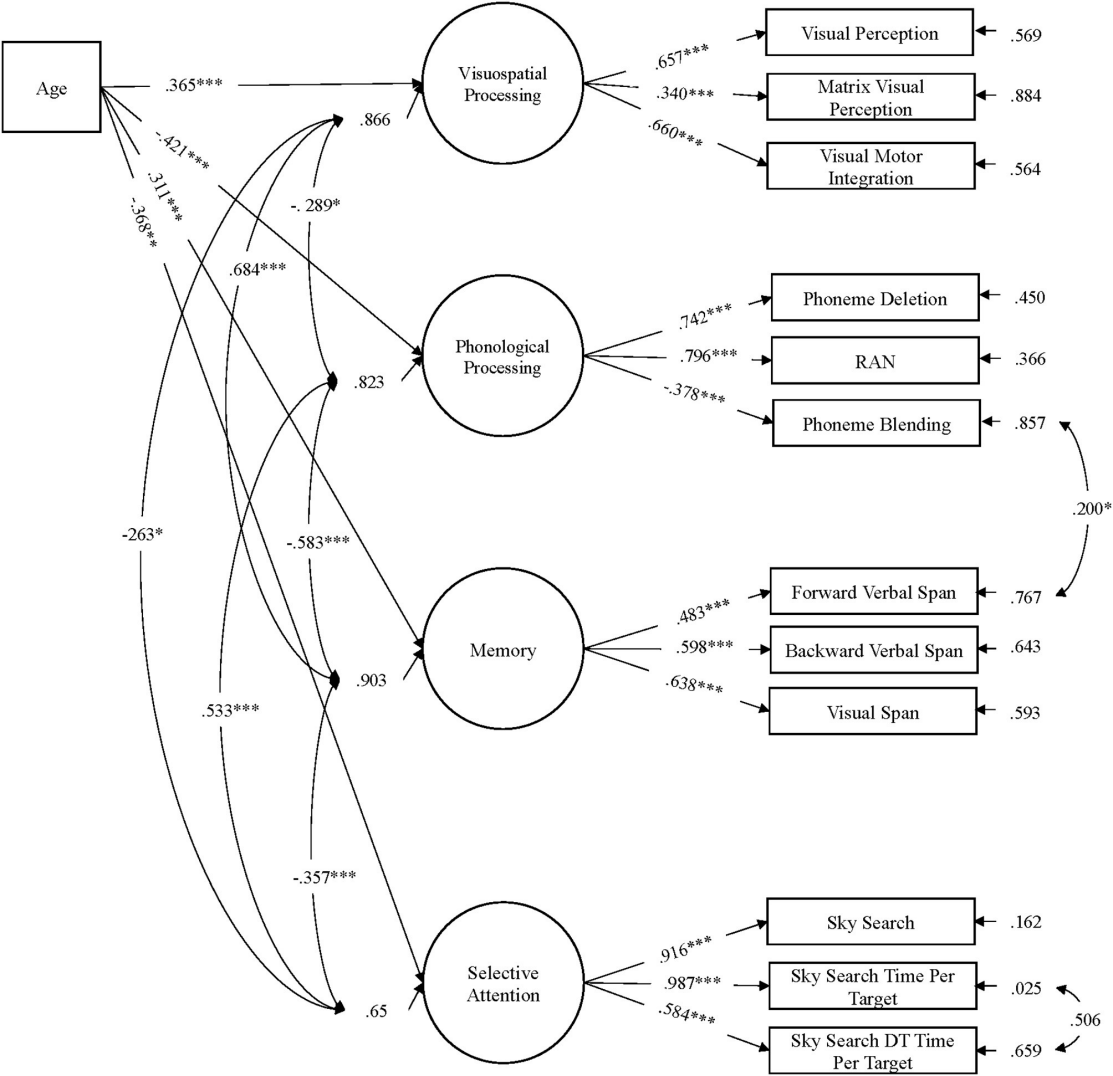
MIMIC path model of age regressed onto the latent factors visuospatial processing, phonological processing, memory, and selective attention (*N* = 153). Standardized parameter estimates are reported. Performance on all latent variables significantly increased with age. ***p < 0.001, **p < 0.01, *p < 0.05.

To account for the effects of age on the latent variables, all four latent variables were regressed onto the age covariate. The MIMIC model produced an acceptable fit, χ^2^(54) = 63.57, *p* = 0.175, RMSEA = 0.034 [90% CI = 0.000, 0.063], SRMR = 0.059, CFI = 0.99, and TLI = 0.98, with significant loadings of all indicators onto their respective constructs (see Figure 1). The inclusion of age into the model did not alter the factor structure or introduce new areas of strain (modification indices) into the model. The small-to-moderate regression paths between age and the latent variables were all significant. Large significant correlations were present between the latent variables of memory and visuospatial processing, memory and phonological processing, and phonological processing and selective attention. All other factor correlations were moderate in size.

#### Group Comparisons

To compare groups, we extracted refined factor scores from the MIMIC model using the regression approach. That is, we used the factor scores of visuospatial processing, phonological processing, memory, and selective attention as DVs. Group comparisons were subjected to 2 (literacy disorder: present vs. absent) × 2 (motor disorder: present vs. absent) ANCOVAs, weighted to account for group size differences. ANCOVAs were initially run to account for group differences in NVIQ (see Table 6). Where NVIQ was not a significant covariate, we re-ran the analyses without NVIQ and report these. This design affords the opportunity to compare profiles between literacy and motor disorders. A significant main effect of either literacy or motor disorder suggests deficit performance in children with the disorder relative to children without the disorder indicating an independent risk factor. Main effects for both literacy and motor disorder would be indicative of deficits in both disorders, and a shared risk factor. In addition, we compared children with isolated and comorbid disorders to elucidate the nature of literacy and motor comorbidity using *post-hoc* oneway AN(C)OVAs. The means and standard deviations for each group, along with the results from group comparisons on each of the factors are reported in Table 6. In addition, the key findings from analyses on each of the factors is described below.

**Table 6:** Means, standard deviations, main effects, covariates, and interactions from the 2 × 2 weighted AN(C)OVAs of factor scores.

	**LD**^****a****^		**MD**^****b****^		**LD+MD**^****c****^		**TD**^****d****^		**Main effect**				**Covariate**			
									**LD**		**MD**		**NVIQ**		**LD** **×** **MD**	
	***M***	***SD***	***M***	***SD***	***M***	***SD***	***M***	***SD***	***F***	**$\eta_{\rho }^{2}$**	***F***	**$\eta_{\rho }^{2}$**	***F***	**$\eta_{\rho }^{2}$**	***F***	**$\eta_{\rho }^{2}$**
Visuospatial	2.62	0.49	2.42^d^	0.41	1.91^ad^	0.43	2.82	0.49	3.34	0.02	7.32^**^	0.05	42.91	0.24	0.62	<0.01
Phonological	−2.96^bd^	0.70	−3.76	0.47	−2.60^bd^	0.72	−3.68	0.47	27.20^***^	0.15	2.65	0.02	–	–	0.45	<0.01
Memory	1.55^d^	0.25	1.53^d^	0.36	1.08^abd^	0.39	1.78	0.34	8.33^**^	0.05	5.98^*^	0.04	7.22	0.05	0.48	<0.01
Selective Attention	−3.46^d^	1.05	−3.77	0.65	−2.83^d^	1.11	−3.88	0.77	8.74^**^	0.06	1.75	0.01	–	–	0.72	<0.01

*LD, literacy disorder; MD, motor disorder; LD+MD, comorbid literacy and motor disorder; TD, typically developing; NVIQ, non-verbal IQ. The covariate, NVIQ, is not reported for phonological speed and literacy analyses because NVIQ was a non-significant covariate and so was dropped from the model. Superscript letters refer to each group (see first row). Superscript letters by means represent significant (at p < 0.05) differences between groups after applying Bonferroni corrections*.

* *p < 0.05*,

** *p < 0.01*,

*** *p < 0.001*.

##### Visuospatial Processing

After controlling for NVIQ, there was a moderate effect of motor disorder status, but no significant effect of literacy disorder status. *Post-hoc* comparisons confirmed that children with motor disorder (MD and LD+MD) had significantly lower visuospatial scores than TD children. There were no significant differences between either MD groups, or between children with LD (in the absence of a motor disorder) and TD controls.

##### Phonological Processing

Children with literacy disorder (LD and LD+MD) performed less well than children without literacy disorder. *Post-hoc* comparisons revealed children with LD and LD+MD performed less well than both TD children and children with MD. No significant differences arose between children with isolated and comorbid LD or between children with isolated MD and TD controls.

##### Memory

Children with LD had poorer memory skill than children without literacy disorder. Similarly, children with MD had poorer memory skills than children without motor disorder. *Post-hoc* comparisons confirmed that all three disorder groups had significantly lower memory skill scores than TD children. Memory skill scores did not differ between children with LD-only and MD-only. However, children with LD+MD had poorer memory skills than either isolated disorder group. When interpreting this finding is it important to note that there is also a lack of significant interaction between literacy and motor disorder status, demonstrating statistical independence of the two disorders. Together, this suggests that the comorbid profile had a combination of deficits that were no different to children with isolated literacy and motor disorders.

##### Selective Attention

Children with LD had lower selective attention scores than children without literacy disorder. *Post-hoc* analyses showed that children with literacy disorder (LD and LD+MD) performed less well than TD children. No significant differences arose between children with LD and LD+MD or between children with MD and TD controls.

## General Discussion

We investigated the relationship between literacy and motor disorders within the context of Pennington's multiple deficit model (MDM). Specifically, in Study 1, we examined prevalence rates of isolated and comorbid literacy and motor difficulties in a community sample to establish whether comorbid literacy and motor difficulties were greater than would be predicted by the base rates of isolated disorders. We found cases of comorbid literacy and motor difficulties to be five times more prevalent than would be expected from the product of isolated disorder base rate estimates, suggesting comorbidity is not the result of chance alone. In Study 2, we examined the relationship between literacy and motor disorders using a subsample of children from Study 1. We aimed to identify potential independent and shared cognitive risk factors of literacy and motor disorders and to compare the profiles of performance on these between children with isolated and comorbid disorders to elucidate the nature of comorbidity between the two disorders. We found phonological processing and selective attention to be independent risk factors for literacy disorders and visuospatial processing to be an independent risk factor for motor disorder. Memory, however, was a shared risk factor for literacy and motor disorders. Comparisons between isolated and comorbid groups revealed children with comorbid literacy and motor disorder to have deficits similar in nature and magnitude to children with isolated literacy and motor disorders.

### Prevalence of Isolated and Comorbid Literacy and Motor Difficulties

The prevalence rates of isolated disorders observed in the present study are considerably lower than those reported in previous studies using clinic and smaller community-based samples to examine comorbid literacy and motor difficulties (Kaplan et al., 1998; Cruddace and Riddell, 2006). However, despite differences in how literacy and motor difficulties were operationalized, the prevalence rates of isolated disorders found here (7% for literacy difficulties and 6% for motor difficulties) corroborate estimates often reported in the literature (e.g., Blank et al., 2012; Snowling, 2013). Such good agreement suggests the current rates are accurate, which is in turn crucial for investigating comorbid disorders because they act as base rates in establishing whether the prevalence of comorbid disorders is greater than would be expected by chance (Caron and Rutter, 1991).

The current prevalence rates of children with comorbid literacy and motor difficulties were also considerably smaller than those reported in previous community (Cruddace and Riddell, 2006) and clinic (Kaplan et al., 1998) samples. Specifically, 3% of the entire sample studied here had comorbid literacy and motor difficulties whereas Cruddace and Riddell (2006)—who also assessed children in community primary schools—identified 13% of children with comorbid profiles. The authors reported relatively high prevalence rates of isolated reading and motor disorders suggesting their sample was not representative of the general population. Indeed, the class teachers in the study noted there was an unexpected number of children with developmental disorders in the classes that were tested. The abnormally high rates of developmental disorders in Cruddace and Riddell's (2006) sample may explain why their estimates of comorbid difficulties were larger than the ones we found in this investigation.

Despite there being little agreement in the exact prevalence rates of comorbid literacy and motor difficulties across studies, all investigations have reported a disproportionately high frequency of comorbid disorders (Kaplan et al., 1998; Cruddace and Riddell, 2006). This corroborates the current findings, where the frequency of children with comorbid literacy and motor difficulties was greater than would be expected by chance alone when using accurate base rates of isolated disorders. Furthermore, the current risk of comorbidity between literacy and motor difficulties is similar to other heterotypic comorbidities found between reading disorder and ADHD (OR = 2.63–5.57; Carroll et al., 2005) and math disorder (OR = 4.1; Landerl and Moll, 2010). Nevertheless, our findings of a relatively high prevalence of comorbidity between literacy and motor difficulties are in accordance with predictions from the MDM and offers some evidence that literacy and motor disorders are likely related.

### Relationship Between Literacy and Motor Disorders

After identifying a high risk of comorbidity between literacy and motor difficulties, we considered the cognitive profiles of children with literacy, motor, and comorbid literacy and motor disorder. In doing so, we re-assessed children on a second, individually administered reclassification battery. As a more in-depth assessment of literacy and motor skills could be carried out, we defined literacy disorder as an impairment in word-level reading (and spelling) skills and motor disorder as an impairment in executing coordinated motor skills. These definitions are closer in classification to the diagnostic labels of dyslexia and DCD.

Group comparisons of factor scores allowed us to search for potential independent and shared cognitive risk factors for literacy and motor disorders. Much of the literature reporting an overlap between literacy and motor disorders had appeared to do so without controlling for comorbid cases in their samples (but see Bellocchi et al., 2017; Biotteau et al., 2017a). In identifying and considering comorbidity separately in the present study, we found independent and shared deficits between the two disorders.

It is often reported that children with motor disorders have deficits in visuospatial processing, with and without a motor component (e.g., Bonifacci, 2004; Tsai and Wu, 2008; Wilson et al., 2013). Such processing deficits have also been reported in children with literacy disorders, although to a much lesser extent (Iversen et al., 2005; Bellocchi et al., 2017). We found a moderate deficit in children with motor disorder, but not in children with literacy disorder, suggesting visuospatial processing is an independent risk factor of motor, but not literacy, disorder. Further analyses (available upon request from the first author) of group performance on the individual tasks visuospatial processing with and without motor components tasks revealed only children with MD to have deficits on these, with larger deficits on the task with a motor component than the task without, in line with Wilson and McKenzie (1998). The absence of evidence of visuospatial processing deficits in our sample of children with literacy disorder contradicts claims that visuospatial deficits may feature in literacy disorders, and instead suggest that such findings may reflect the addition of children with comorbid literacy and motor disorders.

We also examined potential overlap in phonological deficits between literacy and motor disorders. Phonological deficits are widely believed to be a cognitive risk factor of literacy disorder (Pennington and Lefly, 2001; Dandache et al., 2014; Moll et al., 2016), but there are also reports of children with motor disorder performing less well on phonological processing tasks (Dewey et al., 2002; Archibald and Alloway, 2008). As expected, we found large deficits in phonological processing in literacy but not motor disorder. Again, the differences between our findings and those of previous studies could be explained by their lack of control of comorbid cases (e.g., Dewey et al., 2002). Specifically, many previous studies have not controlled for potential comorbid cases and they often reported large variations in phonological skills amongst children with DCD. In the present study, we did not find any significant impairments in phonological skills amongst children with motor disorder, suggesting that findings of phonological deficits in earlier studies reflected the lack of control for comorbid LD cases.

Deficits in memory and attention have also been suggested in both literacy and motor disorders (Cruddace and Riddell, 2006; Alloway, 2007; Swanson et al., 2009). Interestingly, we found the presence of selective attention deficits only in children with word-level literacy disorder, suggesting this is an independent risk factor for dyslexia and not shared between dyslexia and motor disorders. Selective attention deficits in dyslexia have been reported in other studies (e.g., Varvara et al., 2014). In considering the relationship between word-level difficulties and selective attention deficits, it is likely that selective attention deficits on their own are not directly causally related to dyslexia, but rather are distally related to increasing the risk of a child meeting a diagnostic threshold (Hulme and Snowling, 2013; Gathercole et al., 2016).

The lack of selective attention deficits amongst children with motor disorder may appear contradictory at first glance, given the oft reported incidence of attentional difficulties amongst children with DCD (e.g., Dewey et al., 2002). However, the type of attention of interest in this study—selective attention—does not discriminate between children with and without attentional difficulties (see Manly et al., 2001). Therefore, our findings are important as they seem to rule out selective attentional difficulties as a risk factor for isolated and comorbid motor difficulties and LD+MD. However, the impact of attention on the expression of various developmental disorders is evidently complex, and further work should explore this issue.

Consistent with the literature, we found somewhat poorer memory skills in isolated and comorbid disorder groups, suggesting deficits may be shared between these disorders. Further analyses (available on request from the first author) of group performance on the verbal vs. visual memory tests revealed that impairments of verbal memory are a stronger marker for literacy disorder whereas impairments of visual memory are a stronger marker for motor disorder. These findings are in line with previous studies on the differential nature of memory impairments associated with dyslexia and DCD (Maziero et al., 2020). It is important to note, though, that the measure of visual memory used in this study includes a motor component, which limits the strength of the conclusion we can draw about visual memory as a specific marker for motor disorders. To mitigate this potential confound, we used a latent variable approach to reduce variance which may have been related to the motor skills. Nevertheless, we still found children with motor disorders to have poorer memory skills. Future studies should consider how best to disambiguate the influences of memory in different modalities on motor skills in children with DCD.

There is some debate in explaining poor performance on memory tasks in children with developmental disorders (see Gathercole et al., 2016). We take the view that deficits in memory are not directly causally related to the disorder, but rather reflect two, not mutually exclusive, possibilities. The first is that poor performance on memory measures is a downstream consequence of the proximal causal deficit. For example, in dyslexia, poor performance on measures of verbal memory have been attributed to deficits in phonological processing (e.g., McDougall et al., 1994). Similarly, in DCD, visual memory has been attributed to deficits in motor planning (Alloway and Warner, 2008). Another possibility is that memory impairments may be a correlate of literacy and motor disorders, and may not directly cause, but rather act synergistically with proximal causal deficits to increase the likelihood of children meeting a diagnostic threshold (Hulme and Snowling, 2013; Gathercole et al., 2016). The current data—demonstrating poorer performance of children with literacy and/or motor disorders on a measure comprising verbal and visual memory—suggests the latter possibility may be true but does not preclude the former also being true. Further work should examine the nature of memory deficits and their relations to proximal causes of comorbid literacy and motor disorders.

### Nature of Comorbid Literacy and Motor Disorders

Another aim of this study was to examine the profiles of children with comorbid literacy and motor disorders in the light of the three competing hypotheses of the basis of comorbidity (phenocopy, cognitive subtype, and shared etiology). In this group, most children achieved lower non-verbal ability scores. We were careful, however, to ensure these children were not deemed at risk for, nor had received a diagnosis of ID. Furthermore, we controlled (covaried) for group differences in nonverbal ability during the analyses. Children with comorbid literacy and motor disorder performed similarly to children with isolated literacy and isolated motor disorders in all domains. Notably, on memory (a shared risk factor for literacy and motor disorders) children with comorbid literacy and motor disorder had larger deficits than the isolated groups. Moll et al. (2016) found a similar pattern of larger deficits amongst children with comorbid dyslexia and dyscalculia when compared to children with isolated disorders on measures of verbal memory. In both the current study and in Moll et al. (2016), the absence of a statistical interaction between the literacy and motor factor suggests that deficits in the comorbid group reflected deficits in both isolated groups. Taken together, the current findings are most consistent with a shared etiology hypothesis (de Jong et al., 2006) and add to the growing evidence in favor of this hypothesis between dyslexic heterotypic comorbidities (e.g., Gooch et al., 2011; Moll et al., 2016). A shared etiology account proposes that comorbid disorders result from shared genetic origins and is consistent with the MDM account.

It is clear then from the current findings and those from studies of other heterotypic comorbidities at the behavioral and cognitive levels that comorbidity between neurodevelopmental disorders reflects a shared etiology. However, further work on the neural profiles of children with comorbid disorders should be undertaken. Biotteau et al. (2017b) recently examined the behavioral and neural profiles of children with dyslexia, DCD, and comorbid dyslexia and DCD when completing a sequence learning task. The authors found no difference between the groups on task accuracy, consistent with predictions of the shared etiology hypothesis and the MDM. Yet, the fMRI data revealed neural correlates were similar in children with dyslexia and comorbid dyslexia and DCD but different in children with DCD. This suggests children with DCD could have a distinct neural profile to children with comorbid dyslexia and DCD, potentially contradicting predictions from the multiple deficit model. However, such a conclusion requires confirmation from a comparison with typically developing controls. Whilst the juxtaposition of results from behavioral/cognitive and neural profiles should be treated with caution, they raise the valid point that predictions of the MDM should also be tested at the neural level.

The current findings have implications for both researchers and educators. They highlight the potential confounding influence of comorbid cases in research and practice in neurodevelopmental disorders. Researchers and practitioners should be encouraged to screen for additional disorders beyond the disorder of focus. The additive nature of comorbid literacy and motor disorders means that existing tests rather than new comorbid-disorder-specific measures, can be applied in combination to assess comorbidity. Indeed, analysis of the screening battery we used in Study 1 suggests that a battery screening for fine-motor skills was appropriate for group screening class children for motor difficulties. This offers a potential economical and logistical method for identifying motor difficulties among children in large group settings. Further work should examine this possibility closely and consider whether the concomitant use of questionnaires (e.g., DCDQ′07; Wilson et al., 2000) may additionally improve the sensitivity and specificity.

This study investigated the relationship between literacy and motor disorders. It was concerned with exploring and disentangling the high degree of apparent overlap between literacy (e.g., dyslexia) and motor disorders (e.g., DCD), and understanding the nature of comorbidity between the disorders within the context of predictions made by the MDM. In accordance with these predictions, we found a higher rate of comorbidity between literacy and motor disorders, than would be expected by chance. After controlling for comorbidity, it was apparent that literacy and motor disorders were separable disorders with independent—phonological, visuospatial, and selective attention—and shared—memory—deficits. Children with comorbid literacy and motor disorders had deficits that were additive in nature suggesting a shared etiology. Taken together, literacy and motor disorders are two neurodevelopmental disorders that seem to result from independent and shared risk factors that lead to difficulties in literacy and/or motor skills acquisition.

## Data Availability Statement

The datasets presented in this article are not readily available because the data set is new, part of a larger data set, and still being exploited by the authors. The data will be made available in the future. Requests to access the datasets should be directed to Markéta Caravolas, m.caravolas@bangor.ac.uk.

## Ethics Statement

The studies involving human participants were reviewed and approved by Research Ethics Committees of the School of Psychology, Bangor University, and the National Health Service (NHS). Written informed consent to participate in this study was provided by the participants' legal guardian/next of kin.

## Author Contributions

This work was undertaken as part of CD's doctoral studies. CD and MC conceptualized the aims and design of the study. CD performed data collection, scoring, undertook the analysis, and drafted the manuscript. MC supervised the research undertaken, provided guidance on structure and content of the manuscript, and edited each draft version. All authors contributed to the article and approved the submitted version.

## Conflict of Interest

The authors declare that the research was conducted in the absence of any commercial or financial relationships that could be construed as a potential conflict of interest.
