# Supplementary material for: Prevalence and Cognitive Profiles of Children With Comorbid Literacy and Motor Disorders
Source: Front Psychol. 2020 Dec 11;11:573580. doi: 10.3389/fpsyg.2020.573580 (PMC7759613; doi:10.3389/fpsyg.2020.573580)
Supplement: Supplementary file 1 [file Table_1.DOCX]

Supplementary Material

**Supplementary Table 1 | Descriptive statistics of measures for the pooled sample of participants**

|  |  | *M* | *SD* | Range | Reliability |
| --- | --- | --- | --- | --- | --- |
| Literacy | |  |  |  |  |
|  | Word Reading | 38.68 | 8.91 | 18-58 | .93^a^ |
|  | One Minute Word Read | 86.51 | 19.33 | 38-128 | .80^b^ |
|  | One Minute Pseudoword Read | 40.74 | 16.95 | 4-86 |  |
| Motor | |  |  |  |  |
|  | Motor Coordination | 21.71 | 2.57 | 16-26 | .70^a^ |
|  | Lace Threading | 25.71 | 10.07 | 16-111 | .48^b^ |
|  | Balance | 21.73 | 8.54 | 2-30 | .73^b^ |

*Note.* ^a^Cronbach’s alpha. ^b^Correlation coefficient.

**Supplementary Table 2 | Descriptive statistics of visuospatial processing, phonological processing, memory, and selective attention tests as a function of assessment group**

|  |  | | LD | | |  | MD | | |  | LD + MD | | |  | TD | | |
| --- | --- | --- | --- | --- | --- | --- | --- | --- | --- | --- | --- | --- | --- | --- | --- | --- | --- |
|  |  | | *M* | *SD* | *Range* |  | *M* | *SD* | *Range* |  | *M* | *SD* | *Range* |  | *M* | *SD* | *Range* |
| Visuospatial Processing | | |  |  |  |  |  |  |  |  |  |  |  |  |  |  |  |
|  | | Visual Perception | 23.96 | 1.99 | 18-28 |  | 23.65 | 2.27 | 16-29 |  | 21.25 | 2.67 | 16-25 |  | 24.31 | 2.19 | 19-29 |
|  | | Matrix Visual Perception | 17.42 | 1.58 | 10-19 |  | 14.17 | 3.52 | 8-16 |  | 13.5 | 3.63 | 4-18 |  | 16.81 | 2.12 | 10-19 |
|  | | Visual Motor Integration | 19.85 | 3.07 | 13-27 |  | 17.04 | 2.80 | 12-25 |  | 16.45 | 2.09 | 13-20 |  | 20.99 | 2.63 | 15-26 |
| Phonological Processing | | |  |  |  |  |  |  |  |  |  |  |  |  |  |  |  |
|  | Phoneme Deletion | | 142.14 | 43.42 | 70-230 |  | 112.06 | 30.92 | 69-206 |  | 172.52 | 47.86 | 100-274 |  | 107.59 | 34.29 | 58-219 |
|  | RAN | | 26.44 | 6.55 | 17-45 |  | 20.37 | 4.24 | 16-36 |  | 27.62 | 8.68 | 16-50 |  | 19.53 | 3.79 | 12-36 |
|  | Phoneme Blending | | 15.56 | 3.76 | 7-23 |  | 17.38 | 4.75 | 7-23 |  | 14.3 | 4.61 | 4-21 |  | 18.41 | 3.12 | 8-24 |
| Memory | | |  |  |  |  |  |  |  |  |  |  |  |  |  |  |  |
|  | Forward Verbal Span | | 4.59 | 0.97 | 4-8 |  | 4.83 | 0.96 | 3-7 |  | 4.55 | 1.00 | 3-7 |  | 5.37 | 1.13 | 3-8 |
|  | Backward Verbal Span | | 3.26 | 0.76 | 2-5 |  | 3.33 | 0.64 | 2-4 |  | 2.75 | 1.02 | 2-6 |  | 3.52 | 1.00 | 2-8 |
|  | Visual Span | | 26.63 | 3.80 | 20-36 |  | 24.79 | 3.18 | 17-29 |  | 21.65 | 5.82 | 9-31 |  | 26.85 | 3.62 | 19-38 |
| Selective Attention | | |  |  |  |  |  |  |  |  |  |  |  |  |  |  |  |
|  | Sky Search | | 4.84 | 1.31 | 3-8 |  | 4.21 | 1.02 | 2-6 |  | 5.55 | 1.57 | 3-8 |  | 4.08 | 1.16 | 2-8 |
|  | Sky Search TPT | | 5.82 | 1.77 | 2-10 |  | 5.34 | 1.11 | 4-8 |  | 6.91 | 1.90 | 4-11 |  | 5.12 | 1.29 | 3-9 |
|  | Sky Search DT TPT | | 5.63 | 1.17 | 4-8 |  | 6.01 | 1.86 | 3-9 |  | 7.78 | 2.62 | 4-12 |  | 5.35 | 1.46 | 3-8 |
