# Supplementary figures and images for: Prevalence and Cognitive Profiles of Children With Comorbid Literacy and Motor Disorders
Source: Front Psychol. 2020 Dec 11;11:573580. doi: 10.3389/fpsyg.2020.573580 (PMC7759613; doi:10.3389/fpsyg.2020.573580)

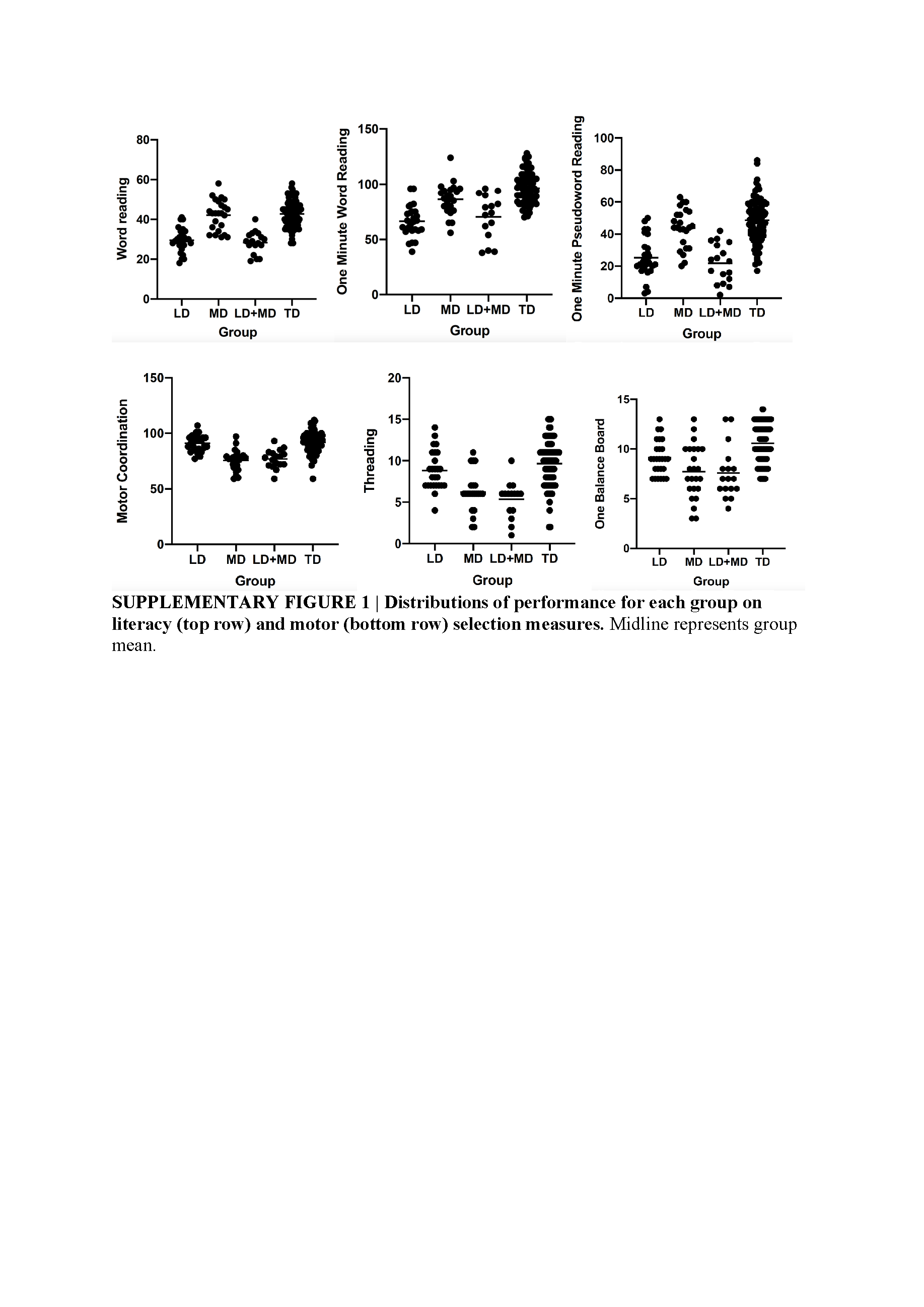

Supplement: Supplementary file 2 [file Image_1.tiff]
